# Comparative Evaluation of the Shear Bond Strength of Self-Adhesive and Glass Ionomer Cement to Dentin After Removal of Hemostatic Agents Using Different Cleansing Protocols: An In Vitro Study

**DOI:** 10.7759/cureus.95030

**Published:** 2025-10-21

**Authors:** Hemashree Namburajan, Mathew Chalakuzhiyil Abraham, Vidhyasankari N, Rajkumar K, Abhinayaa Suthagar, Vishnupriya Venkatasubramanian, Sindhuja Nagarajan

**Affiliations:** 1 Department of Prosthodontics and Crown and Bridge, KSR Institute of Dental Science and Research, Tiruchengode, IND

**Keywords:** aluminium chloride, cleansing protocol, ferric sulfate, glass ionomer cements, self-adhesive resin cement

## Abstract

Background

Hemostatic agents (HA), such as aluminium chloride (AlCl3) and ferric sulfate (FeSO4), can affect the dentin by obstructing adhesive penetration, and reducing the bond strength of self-adhesive resin cements (SARC) and glass ionomer cements (GIC).

Aim

To evaluate and compare the shear bond strength (SBS) of bonding and luting agents (SARC and GIC) to dentin after the removal of AlCl3 and FeSO4, using different cleansing protocols (dentin conditioner (DC) and ethylenediaminetetraacetic acid (EDTA)), and to determine whether the same cleansing protocol can be applied for both bonding and luting procedures.

Methodology

Sixty molar teeth were sectioned and categorized into two HA groups and three cleansing-protocol subgroups. Within each subgroup, specimens were bonded with SARC and GIC. SBS was evaluated, and data were statistically analyzed using independent t-tests, one-way and three-way analysis of variance (ANOVA), with the level of significance established at p < 0.05

Results

For SARC, EDTA yielded the highest SBS after both FeSO4 and AlCl3 contamination, followed by DC and control (p < 0.001). For GIC, DC produced the greatest SBS after AlCl3 contamination (p = 0.042), whereas EDTA was most effective after FeSO4 contamination (p = 0.017). Control groups consistently showed the lowest SBS values.

Conclusion

EDTA is the preferred cleanser for SARC after use of both contaminants, and for GIC following FeSO4 contamination, while DC was preferred for GIC after AlCl3.

## Introduction

Digital dentistry is revolutionizing the field by enabling precise image capture and data visualization. However, margin isolation requires clear visualization, often requiring alternative retraction methods [1].The use of cords has proven more effective than cordless techniques in situations where greater gingival retraction is needed [2]. Indirect restorations like inlays, onlays, veneers, and crowns often bond without rubber dam isolation, but contamination from blood, gingival crevicular fluid, or saliva can deteriorate cement adhesive. Thus, it has been advised for a chemomechanical approach using a cord impregnated with a hemostatic agent, vasoconstrictor, or astringent [3].

Glass ionomer cement (GIC) is widely used as a dental adhesive because of its chemical affinity for tooth structures, sustained fluoride release, caries resistance, biocompatibility with the pulp, and a low thermal expansion coefficient that contributes to improved retention [4]. Self-adhesive resin cements (SARC) are formulated to simplify the luting process by minimizing technique sensitivity and reducing clinical time. These cements can be applied directly to dentin without any surface pretreatments and are suitable for bonding the indirect restorations, including those made from metal alloys, ceramics, and composites [5].

Hemostatic agents (HA) are effective in arresting significant bleeding from capillaries. Aluminium chloride (AlCl3) and ferric sulfate (FeSO4) are preferred, as they cause minimal tissue damage [6]. With their acidic and hydrophilic properties, they can contaminate bonding procedures and cause morphological changes in dentin surfaces. Therefore, the dentin surface must be thoroughly rinsed and cleaned because residual particles can impede resin flow and weaken the bond [7].

Various physical and chemical cleaning methods are suggested for removing HA residues. Physical methods include abrasion with pumice paste, using instruments like excavators, and sandblasting with aluminum oxide particles of varying sizes. Dentin conditioner, phosphoric acid, sodium hypochlorite, chlorhexidine, and ethylenediaminetetraacetic acid (EDTA) are examples of chemical cleaning procedures [8].

It remains unclear if the use of different HA can result in different bond strength values for the same adhesive or dental cement, or if the same cleansing protocol can be used for both luting and bonding procedures [9]. Therefore, it is essential to evaluate the SBS of SARC and GIC to dentin after the removal of hemostatic agents, utilizing different cleansing protocols.

## Materials and methods

The study was conducted at the Department of Prosthodontics and Crown & Bridge, for sample preparation (IEC ref no: 375/KSRIDSR/IEC/2023). The sample size was determined based on the study by Duygu Saraç [10], with an effect size of 0.42, a significance level (α) of 0.05, and a statistical power (β) of 0.80. G*Power software was used for calculation (version 3.1.9.3 for Macintosh; Heinrich Heine University, Düsseldorf, Germany), indicating that a minimum of 60 samples was required.

**Figure 1:**
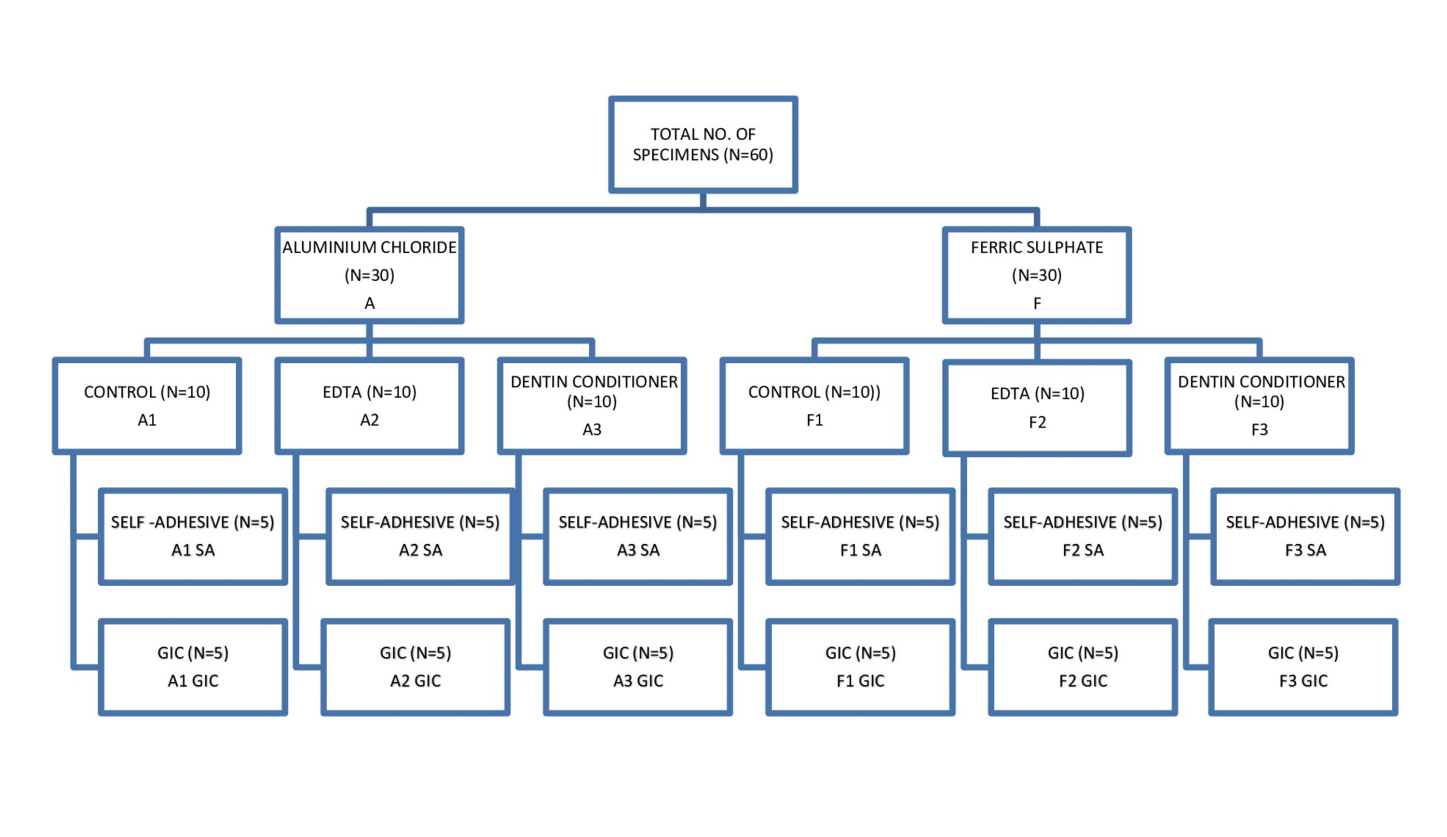
Grouping

Sixty extracted human molars (intact and non-carious) were selected. They were sectioned and categorized into two HA groups and three cleansing-protocol subgroups (Figure 1). All teeth were manually scaled to eliminate soft debris, then immersed in a 1% hydrogen peroxide solution for 24 hours, followed by thorough rinsing with distilled water [4]. During and between experimental procedures, the teeth were stored in a 10% formalin solution to prevent dehydration and maintain structural integrity. The crowns of the teeth were sectioned longitudinally at the level of the central fossa in the mesiodistal direction using a low-speed diamond disk with copious water cooling [11]. Each crown was embedded in acrylic within a polyvinyl chloride (PVC) cylinder (15 mm × 25 mm), exposing flat occlusal dentin. For finishing, 600‐grit paper was used for 30 s to create a standardized smear layer [12].

Each sample in Groups A and F received a 5-minute application of either 25% aluminum chloride (Viscostat Clear, Ultradent, Utah, US) or 20% ferric sulfate (Viscostat, Ultradent). After that, the specimens were rinsed for 20 seconds at a distance of 10 mm under a water spray with a pressure of 2065-2585 mmHg [13].

Cleansing protocols

For each group, different cleansing protocols were employed after hemostatic agent application.

Control (A1, F1): Rinse with water spray (2065-2585 mmHg) for 10 seconds [14].

EDTA (A2, F2): Scrub with 17% EDTA for 60 seconds using an applicator tip, then rinse with distilled water for 30 seconds without desiccation [14].

Dentin conditioner (A3, F3): Scrub with 10% polyacrylic acid for 20 seconds, then rinse with distilled water for 30 seconds without desiccation [15].

Bonding procedures

All bonding was performed within four hours of specimen preparation. A transparent Teflon mold (3 mm internal diameter × 3 mm height) was centered on each dentin surface to standardize the bonding area.

Self‐adhesive resin cement (RelyX U200, 3M ESPE, Minnesota, US): SARC was injected into the mold and light‐cured with an LED unit (1200 mW/cm²) for 30 seconds. The mold was removed, and any excess cement was trimmed.

Glass ionomer cement (GC Gold Label I / Fuji I; GC India Dental PVT. LTD., Telengana, India): Powder and liquid were mixed for 20 sec to a homogeneous paste, placed into the mold, allowed to set undisturbed for 4-5 mins, and then excess was removed. The final set occurred at approximately 7 minutes post‐mixing.

To replicate intraoral conditions, the specimens were kept at 37 °C in distilled water for 24 hours with 100% humidity after bonding to the dentinal surface (ISO 29022:2013(E)).On a universal testing machine (Instron 3345, Massachusetts, USA), each specimen was placed in a specially designed jig, with a chisel-shaped blade positioned at the dentin-cement interface. Until failure, a shear force was applied at a crosshead speed of 1 mm/min. The maximum load (N) at debonding was recorded. Shear bond strength (MPa) was calculated as: Shear Bond Strength (MPa) = Maximum Load (N) / Bonded Area (7.07 mm²).

Results were expressed in MPa for each specimen.

## Results

Data regarding shear bond strength (N) of the sectioned teeth cemented with either self-adhesive or glass ionomer cement after being initially treated with two different hemostatic agents (aluminum chloride and ferric sulfate), followed by three different cleansing agents (water, EDTA, and dentin conditioner), were analyzed. Normality of the data was assessed using the Kolmogorov-Smirnov test, which confirmed a normal distribution. Descriptive statistics, including mean, standard deviation, 95% confidence intervals, and the range (minimum and maximum values), were calculated. Intergroup comparisons were performed using independent t-tests and one-way analysis of variance (ANOVA), followed by Tukey’s honest significant difference (HSD) test for post-hoc analysis. A p-value of less than 0.05 was considered statistically significant.

The mean shear bond strength of SARC after the use of various cleansing agents for the removal of aluminum chloride shows that the highest shear bond forces were in the EDTA group, followed by Dentin conditioner and control, and the mean difference between these cleansing agents was statistically significant (p = 0.000) (Table 1).

**Table 1:** Intergroup comparison of shear bond strength for various cleansing agents after aluminium chloride removal using self-adhesive and glass ionomer cements *Statistically significant (p<0.05); SBS – Shear bond strength, SA – Self-adhesive cement, GIC – Glass ionomer cement, df – Degrees of freedom, Values represent shear bond strength (MPa)

SBS in the Aluminium Chloride Group		n	Mean ± SD	Std. Error	95% Confidence Interval for Mean		Minimum	Maximum
					Lower Bound	Upper Bound		
SA	Control	5	36.155 ± 12.140	5.429	21.08	51.23	20.866	54.23
	EDTA	5	120.421 ± 18.901	8.452	96.952	143.89	99.64	147.228
	Dentin Conditioner	5	99.334 ± 19.342	8.65	75.317	123.35	80.864	128.491
GIC	Control	5	21.899 ± 6.057	2.709	14.377	29.421	13.047	28.369
	EDTA	5	29.922 ± 6.452	2.885	21.909	37.934	19.041	35.159
	Dentin Conditioner	5	33.742 ± 8.244	3.686	23.506	43.979	20.463	41.439

Whereas, the GIC cement showed the highest mean shear bond forces after the use of dentin conditioner, followed by EDTA and control, and the mean difference in shear bond forces of GIC between these cleansing agents after the removal of AlCl3 was statistically not significant (p = 0.054).

The mean SBS of SARC after the use of various cleansing agents for the removal of FeSO4 had the highest shear bond forces in the EDTA group, followed by the dentin conditioner group and the control, and the mean difference between these cleansing agents was statistically significant (p = 0.000) (Table 2).

**Table 2:** Intergroup comparison of shear bond strength for various cleansing agents after ferric sulfate removal using self-adhesive and glass ionomer cements Data shown as mean ± SD (MPa), n = 5; significance set at p < 0.05

Descriptives:		n	Mean ± SD	Std. Error	95% Confidence Interval for Mean		Minimum	Maximum
SBS in the Ferric Sulfate Group					Lower Bound	Upper Bound		
SA	Control	5	57.677 ± 23.803	10.645	28.121	87.233	35.973	88.624
	EDTA	5	134.579 ± 20.192	9.03	109.506	159.652	104.918	154.115
	Dentin Conditioner	5	124.946 ± 19.064	8.525	101.274	148.617	97.596	149.383
GIC	Control	5	16.808 ± 8.607	3.849	6.12	27.495	7.779	29.941
	EDTA	5	34.882 ± 13.184	5.896	18.511	51.253	21.16	50.454
	Dentin Conditioner	5	16.384 ± 6.3776	2.852	8.465	24.303	10.232	25.006

However, the GIC cement had the highest mean shear bond forces after the use of EDTA, followed by control and dentin conditioner, and the mean difference in shear bond forces of GIC between these cleansing agents after the removal of FeSO4 was statistically significant (p = 0.017).

## Discussion

Hemostatic agents, like AlCl3 and FeSO4, are used in dentistry for restorative and prosthodontic procedures. Hemostatic agents, being hydrophilic, and their contamination can compromise the bond strength of adhesives to tooth structures, increasing the risk of microleakage, caries recurrence, and treatment failure [8]. This study evaluates two cleansing agents, EDTA and dentin conditioner, for the removal of AlCl₃ and FeSO4 to determine their effectiveness in restoring bond strength after contamination.

Influence of cleansing agents on the SBS of SARC

**Figure 2:**
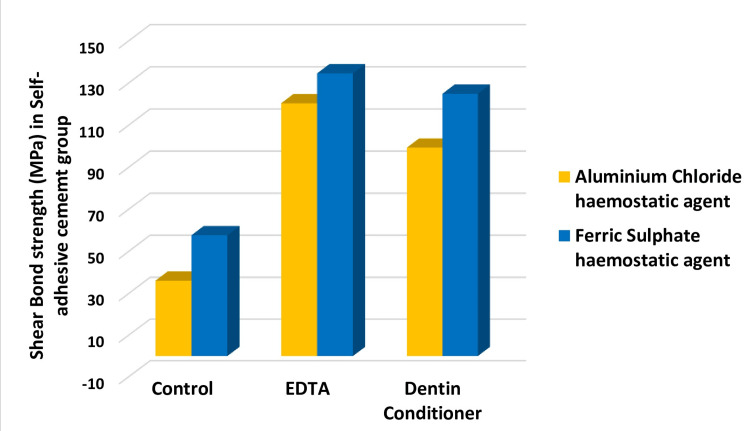
Shear bond strength (N) based on self-adhesive cement

Figure 2 shows the influence of cleansing agents on the SBS of SARC.

After contamination with FeSO4 and AlCl3, the highest mean shear bond strength for SARC was observed when EDTA was used as a cleansing agent. The enhanced bonding ability of EDTA-treated samples can be attributed to its dual action as a chelating and mild demineralizing agent. Our results suggest that EDTA consistently yielded the highest SBS values across both FeSO4- and AlCl3-contaminated groups. This superior performance aligns with findings by Youm et al. (2012), who reported that mild etchants, such as EDTA, significantly improved microtensile bond strength (µTBS) in SARC by eliminating smear layers and contaminants [15].

Dentin conditioner, which contains polyacrylic acid (PAA), also demonstrated substantial efficacy in improving bond strength following hemostatic agent contamination. Pavan et al. (2010) noted that PAA pretreatment enhanced bonding by either modifying the smear layer to create a calcium- and phosphate-rich intermediate layer or completely removing it to expose a collagen-rich bonding zone. This intermediate layer may facilitate chemical interactions with self-adhesive cement monomers, such as phosphoric acid methacrylates, ultimately improving bond strength [16].

Conversely, the control group exhibited significantly lower SBS values in both FeSO4 and AlCl3 contamination scenarios, emphasizing the detrimental effect of residual hemostatic agents on dentin bonding. The inability of self-etching monomers to remove contaminants adequately further contributes to compromised bond strength.

Influence of cleansing agents on the SBS of glass ionomer cement

**Figure 3:**
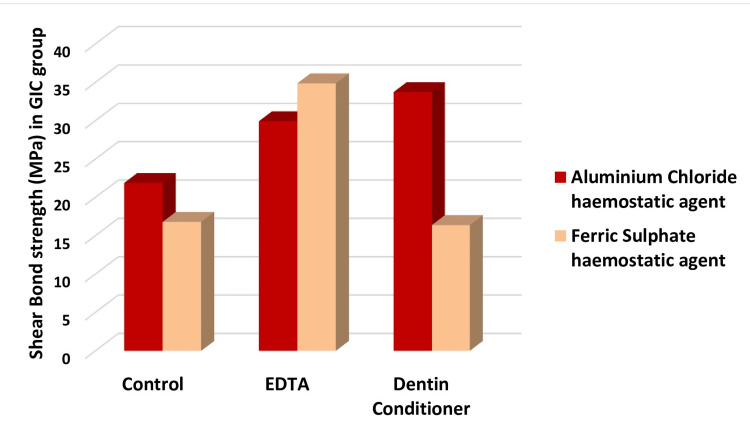
Shear bond strength (N) based on GIC GIC – Glass ionomer cement

Figure 3 depicts the influence of cleansing agents on the SBS of glass ionomer cement.

A comprehensive analysis of the data reveals that EDTA consistently yielded the highest values following FeSO4 contamination, whereas dentin conditioner proved more effective for AlCl3 contamination. The effectiveness of dentin conditioner can be attributed to its primary component, PAA, which significantly improves bond strength after hemostatic agent contamination. PAA facilitates GIC adhesion by removing the smear layer, enhancing surface wettability, and promoting chemical interactions with calcium ions in hydroxyapatite [17].

Supporting these findings, Glasspoole et al. emphasized that PAA irreversibly adsorbs onto hydroxyapatite and establishes ionic bonds with calcium and carboxyl groups [18].This observation aligns with the present study, which found that PAA-based conditioners significantly improved the SBS of GIC to dentin.

EDTA significantly enhanced SBS after ferric sulfate contamination (p = 0.017). These results align with Fagundes et al.'s findings that EDTA facilitates smear layer removal while maintaining the integrity of the collagen network, thereby enhancing the chemical bonding between glass ionomer cement and dentin [16]. Similarly, Poggio et al. found that PAA conditions the dentin surface effectively by cleansing it without fully opening the dentinal tubules, making it the preferred conditioner for GIC bonding [19].

In the case of AlCl3 contamination, dentin conditioner achieved the highest SBS, followed by EDTA, with both significantly outperforming the control (p = 0.042). However, when exposed to FeSO4 contamination, SBS values for dentin conditioner were significantly lower. This can be attributed to FeSO4's interaction with PAA, leading to the formation of insoluble complexes that hinder bonding. In contrast, AlCl3 contamination appears less disruptive, allowing PAA-based conditioners to be more effective. The presence of ferric sulfate (Fe³⁺) interferes with PAA bonding by forming insoluble ferric-polyacrylate complexes while also leaving precipitates of ferric oxide (Fe₂O₃) and ferric hydroxide (Fe(OH)₃), which may obstruct proper adhesion. Additionally, the acidic nature of PAA can accelerate precipitation, leading to surface contamination and reduced bond strength.

The role of PAA in enhancing adhesion was further reinforced by Poggio et al., who demonstrated that PAA increases wettability and ion exchange at the dentin surface, thereby optimizing bond strength [19]. These findings align with those of Romina Mazaheri et al., who concluded that conditioners such as 20% acrylic acid and 17% EDTA reduce microleakage and improve bond strength by eliminating debris and partially demineralizing the surface, thereby facilitating better micromechanical and chemical interactions [20].

The limitation of this study is that it was conducted in a controlled in vitro setting, which does not fully replicate the complex oral environment and may therefore influence clinical outcomes that were not accounted for in the study. The uncontaminated group was not taken as the “control” group because water rinsing after contamination was selected instead, representing the most common initial clinical step following hemostatic agent application in practice. Additionally, the study focused on short-term bond strength assessment without evaluating long-term degradation due to factors such as water absorption, thermal cycling, or mechanical fatigue.

Future studies should include a wider range of luting agents and larger, more diverse sample populations to enhance generalizability. Incorporating microtensile and fatigue testing, as well as evaluations of microleakage, marginal integrity, and long-term bond stability, will provide a more comprehensive assessment of bonding performance under simulated clinical conditions.

## Conclusions

Within the limitations of this in vitro study, it can be concluded that the selection of an appropriate cleansing protocol significantly influences the bond strength of SARC and GIC to dentin contaminated with HA. For self-adhesive resin cement (SARC), EDTA was the most effective cleanser for both AlCl₃ and FeSO₄ contamination, showing superior bond strength compared to dentin conditioner. In contrast, for GIC, EDTA was more effective after FeSO₄ contamination, while dentin conditioner performed better following AlCl₃ contamination. However, dentin conditioner should not be used with FeSO₄, as it significantly reduces bond strength due to the formation of insoluble complexes and interferes with adhesion.

The control group (without cleansing) consistently had the lowest bond strength, confirming the negative impact of hemostatic agents.
